# Characterization of the Impaired Glucose Homeostasis Produced in C57BL/6 Mice by Chronic Exposure to Arsenic and High-Fat Diet

**DOI:** 10.1289/ehp.1003324

**Published:** 2011-05-18

**Authors:** David S. Paul, Felecia S. Walton, R. Jesse Saunders, Miroslav Stýblo

**Affiliations:** Department of Nutrition, University of North Carolina–Chapel Hill, Chapel Hill, North Carolina, USA

**Keywords:** arsenic, diabetes, diet-induced obesity, glucose intolerance, high-fat diet, insulin resistance

## Abstract

Background: Type 2 diabetes is characterized by glucose intolerance and insulin resistance. Obesity is the leading cause of type 2 diabetes. Growing evidence suggests that chronic exposure to inorganic arsenic (iAs) also produces symptoms consistent with diabetes. Thus, iAs exposure may further increase the risk of diabetes in obese individuals.

Objectives: Our goal was to characterize diabetogenic effects of iAs exposure and high-fat diet (HFD) in weaned C57BL/6 mice.

Methods: Mice were fed HFD or low-fat diet (LFD) while exposed to iAs in drinking water (25 or 50 ppm As) for 20 weeks; control HFD and LFD mice drank deionized water. Body mass and adiposity were monitored throughout the study. We measured glucose and insulin levels in fasting blood and in blood collected during oral glucose tolerance tests (OGTT) to evaluate the diabetogenic effects of the treatment.

Results: Control mice fed HFD accumulated more fat, had higher fasting blood glucose, and were more insulin resistant than were control LFD mice. However, these diabetes indicators decreased with iAs intake in a dose-dependent manner. OGTT showed impaired glucose tolerance for both control and iAs-treated HFD mice compared with respective LFD mice. Notably, glucose intolerance was more pronounced in HFD mice treated with iAs despite a significant decrease in adiposity, fasting blood glucose, and insulin resistance.

Conclusions: Our data suggest that iAs exposure acts synergistically with HFD-induced obesity in producing glucose intolerance. However, mechanisms of the diabetogenic effects of iAs exposure may differ from the mechanisms associated with the obesity-induced type 2 diabetes.

Inorganic As (iAs) is one of the most potent environmental carcinogens [International Agency for Research on Cancer (IARC) 1987]. However, chronic exposures to iAs have also been associated with various noncancerous diseases, including diabetes mellitus. Increased risks of diabetes have been reported in populations exposed to high levels of iAs in drinking water and among workers exposed to iAs in occupational settings (Navas-Acien et al. 2006). Although results of epidemiologic studies  examining effects of low exposures to iAs have been inconclusive, laboratory research has shown that exposures to iAs can produce effects that are consistent with diabetes (Navas-Acien et al. 2006; Paul et al. 2007b).

We previously examined associations between iAs exposure and diabetes both in population-based studies and in laboratory studies, using tissue culture and animal models. Our laboratory studies have focused on effects of iAs and its metabolites on the insulin-activated signal transduction pathway that regulates the insulin-dependent glucose uptake in peripheral tissues. We found that trivalent metabolites of iAs—arsenite (iAs^III^), methylarsonite (MAs^III^), and dimethylarsinite (DMAs^III^)—inhibit insulin signaling and insulin-stimulated glucose uptake by cultured 3T3-L1 adipocytes at concentrations that do not affect cell viability (Walton et al. 2004). Specifically, iAs^III^ and MAs^III^ inhibited insulin-dependent phosphorylation of protein kinase B (PKB/Akt), thus preventing translocation of GLUT4 (insulin-sensitive glucose transporter) from the perinuclear compartment of adipocytes to the plasma membrane (Paul et al. 2007a). In contrast, DMAs^III^ inhibited GLUT4 translocation by interfering with signaling steps downstream from PKB/Akt.  We also examined diabetogenic effects of iAs exposure in mice (Paul et al. 2007b). In that study, weanling male C57BL/6 mice fed a regular laboratory chow drank for 8 weeks either deionized water (diH_2_O) or diH_2_O with addition of iAs^III^ at concentrations of 25 or 50 ppm As. Intraperitoneal glucose tolerance tests revealed impaired glucose tolerance in mice exposed to 50 ppm As but not in mice drinking diH_2_O with 25 ppm As or pure diH_2_O (Paul et al. 2007b). Notably, the average concentration of total speciated As in livers from mice in the 50-ppm group was comparable to the highest concentration of total As reported in livers of residents of Bangladesh who had consumed water with order of magnitude lower levels of iAs (Mazumder 2005). These data suggest that mice are less susceptible than humans to the diabetogenic effects of chronic exposure to iAs, possibly due to a more efficient clearance of iAs or its metabolites from target tissues, and that concentrations of iAs higher than those found in drinking water should be used in laboratory studies to reproduce the diabetogenic effects of iAs exposure reported in human populations.

In the present study, we examined interactions of iAs exposure with obesity, the leading cause of diabetes worldwide. Our results suggest that the key characteristics of diabetes produced in mice by an obesogenic diet combined with chronic exposure to iAs [normal fasting blood glucose (FBG) and normal fasting serum insulin (FSI) levels with pronounced glucose intolerance] differ from those described for type 2 diabetes. Thus, iAs exposure may target tissues or regulatory mechanisms that are not typically associated with type 2 diabetes.

## Materials and Methods

*Chemicals.* Sodium arsenite, sodium salt (99% pure), was purchased from Sigma-Aldrich (St. Louis, MO). Ultrapure phosphoric acid was obtained from J.T. Baker (Phillipsburg, NJ). Sodium arsenate (iAs^V^), sodium salt (96%; Sigma-Aldrich); methylarsonate (MAs^V^), disodium salt (98%; Chem Service, West Chester, PA); and dimethylarsinic acid (DMAs^V^; 98%; Strem Chemicals, Inc., Newburyport, MA) were used as standards for speciation analysis of As in mouse tissues. All other chemicals were of the highest grade commercially available.

*Mice.* Four-week-old male weanling C57BL/6 mice were obtained from the Jackson Laboratory (Bar Harbor, ME) and housed in the University of North Carolina–Chapel Hill (UNC-CH) Animal Facility, which is fully accredited by the American Association for Accreditation of Laboratory Animal Care. Mice were housed five per cage in polycarbonate cages with corncob bedding in controlled conditions (12-hr light/dark cycle, 22 ± 1°C, and 50 ± 10% humidity). Mice were fed a low-fat diet (LFD; 11% fat) or a high-fat diet (HFD; 58% fat; both from Research Diets, Inc., Brunswick, NJ) and drank either diH_2_O or diH_2_O plus iAs^III^ (25 or 50 ppm As). Water containing iAs^III^ was freshly prepared every 3–4 days to minimize oxidation of iAs^III^ to iAs^V^. Water and food consumption and body mass were monitored in all exposure groups every week. Body composition was measured biweekly at the UNC-CH Nutrition Obesity Research Core, using EchoMRI-100 (EchoMRI, Houston, TX). The animals were treated humanely and with regard for alleviation of suffering. All procedures involving mice were approved by the UNC-CH Institutional Animal Care and Use Committee.

*Oral glucose tolerance test (OGTT) and analyses of blood glucose and insulin.* After 20 weeks, both control and iAs-treated LFD and HFD mice were fasted overnight before administration of OGTT. d-Glucose (Sigma) was dissolved in diH_2_O and orally administered to the fasted mice (2 g/kg of body weight) using a 20-gauge stainless steel gavage feeding needle (Fisher Scientific, Waltham, MA). Samples of whole blood (2–3 μL each) were collected from a tail-clip bleed immediately before and 15, 30, 60, 90, and 120 min after glucose administration. Blood glucose levels were measured using a Freestyle Glucose Monitoring System (Abbott Laboratories, Abbott Park, IL). Additional samples of whole venous blood (100 μL) were collected from tails immediately before and 15 min after glucose administration for determination of serum insulin levels. The blood was allowed to clot on ice for 15 min and then centrifuged (200 × *g*) at 4°C for 10 min.

Serum was analyzed using the Rat/Mouse Insulin ELISA kit according to the manufacturer’s protocol (Millipore, Billerica, MA). We used the FBG and FSI concentrations to calculate the homeostasis model assessment–insulin resistance (HOMA-IR) value:

HOMA-IR =  [FSI (in microunits per milliliter)  × FBG (in millimoles per liter)]  ÷ 22.5.

The blood glucose and serum insulin levels recorded during OGTT were used to evaluate glucose tolerance and insulin response to glucose challenge, respectively. After OGTT, mice were returned to their cages and treatment continued for 7–10 days before necropsy.

*Blood and tissue collection at necropsy.* Whole blood samples were collected by submandibular bleeds, and mice were sacrificed by cervical dislocation. Hematocrit (a marker of dehydration) was determined in samples of fresh blood (~ 100 μL) using 40-mm heparin-coated capillary tubes. Capillary tubes were centrifuged at 12,000 rpm in a microhematocrit centrifuge (Unico, Dayton NJ) for 15 min, and the percentage of red blood cells was recorded for each mouse. Serum was isolated from submandibular blood as described above. Liver, inguinal adipose tissue, quadriceps, and pancreas were collected during necropsy and snap frozen in liquid nitrogen. Serum and the tissue samples were stored at –80°C until analysis.

*Analyses of hepatic and serum triacylglycerol.* We measured triacylglycerol (TAG) contents in serum and in liver homogenates spectrophotometrically (at 490 nm) after a two-step extraction in a chloroform/methanol (2:1) mixture and in pure chloroform, using a Stanbio Enzymatic Triglyceride Kit (Stanbio, Boerne, TX), following the manufacturer’s instructions.

*Speciation analysis of As in tissues.* For analysis of iAs metabolites, 10% (wt/vol) tissue homogenates were prepared in diH_2_O and digested in ultrapure phosphoric acid. Concentrations of As species were determined in digested homogenates by hydride generation–cryotrapping–atomic absorption spectrometry (HG-CT-AAS), following previously described procedures (Hernández-Zavala et al. 2008). This method detects and quantifies total iAs (iAs^III^ + iAs^V^), total MAs (MAs^III^ + MAs^V^), and total DMAs (DMAs^III^ + DMAs^V^).

*Statistical analysis.* Results of the analyses are expressed as mean ± SE for each treatment group (*n* = 6–10). Effects of diet and iAs exposure, as well as interactions between diet and iAs exposure, were analyzed by analysis of covariance. Differences between the treatment groups were evaluated by one-way analysis of variance with Tukey or Bonferroni multiple-comparison posttests. All statistical analyses were performed using Graphpad Prism (version 5.0; GraphPad Software, San Diego, CA). Differences among means with *p* < 0.05 were considered statistically significant.

## Results

**Figure 1:**
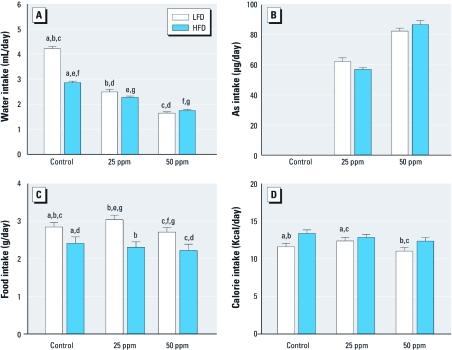
Intakes of water (*A*), As (*B*), food (*C*), and calories (*D*) by control mice or mice exposed to 25 or 50 ppm As and fed LFD or HFD (mean ± SE; *n* = 38 for *A* and *B*; *n* = 17 for *C* and *D*). Values marked with the same letters are statistically significantly different: (*A*) for a–g, *p* < 0.001; (*C*) for d and f, *p* < 0.01; and for a–c and e, *p* < 0.001; (*D*), for b, *p* < 0.01; and for a and c, *p* < 0.001.

*Water, As, food, and calorie intakes.* The daily water, food, and calorie intakes were significantly affected by type of diet and iAs exposure (Figure 1). Control mice on HFD consumed significantly less water than did control mice on LFD (2.86 and 4.21 mL/day, respectively). Consistent with our previous results (Paul et al. 2007b), water intake decreased in both groups exposed to iAs (Figure 1A); LFD and HFD mice exposed to 25 ppm As drank 2.49 and 2.28 mL/day compared with mice in the respective 50-ppm groups that drank 1.68 and 1.74 mL/day. We used hematocrits to characterize the hydration status of the mice in all treatment groups [see Supplemental Material, Figure 1 (doi:10.1289/ehp.1003324)]. We found no significant differences, suggesting that the lower water intakes associated with consumption of HFD or iAs treatment did not cause dehydration. The average daily As intakes were estimated from water intake. Mice on LFD and HFD exposed to 25 ppm As ingested 62.2 and 57.1 μg/day As, respectively; mice on LFD and HFD exposed to 50 ppm As ingested 82.5 and 86.8 μg/day As, respectively (Figure 1B). In general, mice on LFD consumed more food than did mice on HFD (Figure 1C): mice in the LFD groups consumed 2.85 g/day (controls), 3.04 g/day (25 ppm As), and 2.71 g/day (50 ppm As) compared with mice in the HFD groups, which consumed 2.41 g/day (controls), 2.31 g/day (25 ppm As), and 2.22 g/day (50 ppm As). We estimated calorie intakes for mice in the treatment groups using food intake data and caloric densities of LFD and HFD (Figure 1D). Exposure to iAs led to a small decrease in calorie intake in the HFD group: 13.4 calories/day for controls, 12.9 calories/day  for 25 ppm As, and 12.4 calories/day for 50 ppm As. However, these differences were not statistically significant.

**Figure 2:**
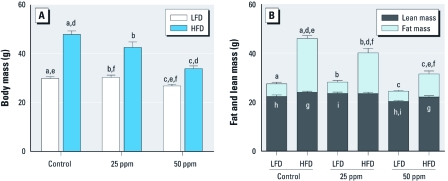
Body mass and body composition (mean ± SE) of control mice or mice exposed to 25 or 50 ppm As and fed LFD or HFD (*n* = 9–10). (*A*) Total body mass. (*B*) Lean and fat mass. Values marked with the same letters are statistically significantly different: (*A*) for e, *p* < 0.05; for c and f, *p* < 0.01; and for a, b, and d, *p* < 0.001; (*B*) for d, g, and h, *p* < 0.05; for c and f, *p* < 0.01; and for a, b, e, and i, *p* < 0.001.

*Body mass and composition.* Figure 2 shows body mass and body composition for control and iAs-treated mice fed LFD and HFD. As expected, control mice fed HFD for 20 weeks accumulated significantly more fat and weighed more than the control mice on LFD. However, iAs exposure decreased both the HFD-induced weight gain and fat accumulation. Specifically, the mice fed HFD and exposed to 25 and 50 ppm As weighed less (42.6 and 33.9 g) and had less fat mass (16.7 and 9.4 g) than did the HFD controls (47.9 g body mass and 22.0 g fat mass). The differences in both total body mass and fat mass were statistically significant. Notably, iAs exposure had no statistically significant effects on lean mass of mice in the HFD group. iAs exposure had no effect on fat mass in LFD mice; however, lean mass decreased by 9% in LFD mice exposed to 50 ppm As compared with controls (*p* < 0.05).

**Figure 3:**
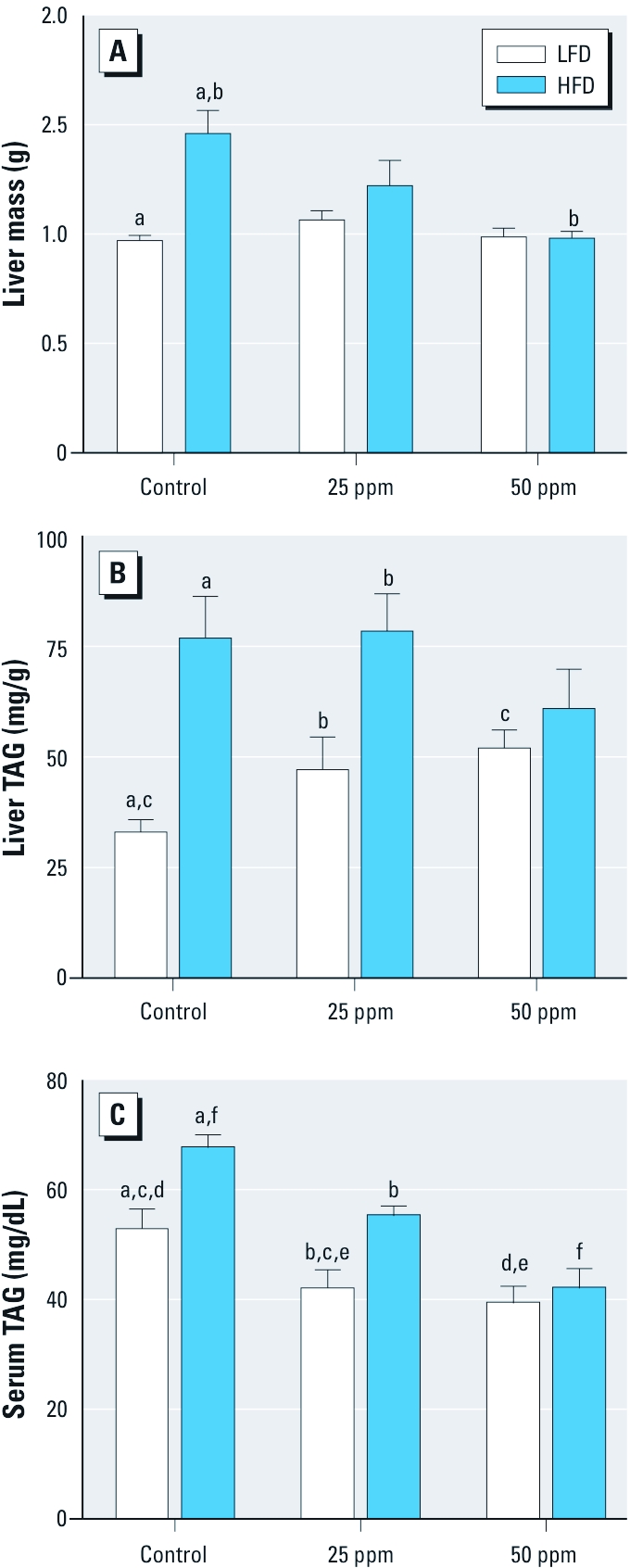
Markers of adiposity (mean ± SE) in control mice or mice exposed to 25 or 50 ppm As and fed LFD or HFD (*n* = 9–10). (*A*) Liver mass. (*B*) Liver TAG. (*C*) Serum TAG. Values marked with the same letters are statistically significantly different: (*A*) for b, *p* < 0.01; and for a, *p* < 0.001; (*B*) for c, *p* < 0.05; for b, *p* < 0.01; and for a, *p* < 0.001; (*C*) for b and f, *p* < 0.05; for a, c, and e, *p* < 0.01; and for d, *p* < 0.001.

*Liver adiposity and serum TAG concentration.* The average total liver mass was significantly greater in control mice on HFD compared with LFD controls (Figure 3A). However, iAs exposure substantially reduced or eliminated the difference in liver mass between HFD and LFD mice. To determine whether these effects are associated with differences in fat accumulation, we compared TAG concentrations in the livers of mice in all treatment groups (Figure 3B). The average TAG concentration was > 2-fold higher in the livers of HFD controls compared with LFD controls. The difference in the hepatic TAG concentrations remained statistically significant for HFD and LFD mice exposed to 25 ppm As but not for the mice exposed to 50 ppm As. This was in part because the exposure to iAs increased TAG concentration in a concentration-dependent manner in livers of mice fed LFD; we noted a statistically significant elevation in liver TAG for LFD mice exposed to 50 ppm As compared with LFD controls (*p* < 0.05). We also measured TAG concentrations in serum of fasted mice from all treatment groups (Figure 3C). In general, consumption of HFD was associated with higher serum TAG levels. However, iAs exposure lowered serum TAG in the LFD groups and particularly the HFD groups in a concentration-dependent manner. As a result, we observed no statistically significant difference in serum TAG levels between the LFD and HFD mice exposed to 50 ppm As.

**Figure 4:**
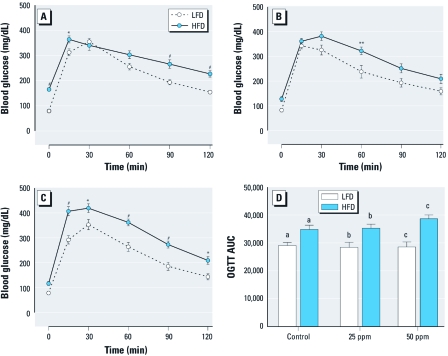
OGTT results (mean ± SE) for control mice (*A*) and mice exposed to 25 ppm As (*B*) or 50 ppm As (*C*) and fed LFD or HFD (*n* = 9–10). Average FBG values correspond to the values at time 0. (*D*) AUCs for mice fed LFD or HFD. *p < 0.05, **p < 0.01, and ^#^*p* < 0.001, compared with LFD mice. Values marked with the same letters are statistically significantly different: (*D*) for a, *p* < 0.05; for b, *p* < 0.01; and for c, *p* < 0.001.

*Glucose tolerance.* We used FBG and 2-hr OGTT to evaluate glucose homeostasis and tolerance (Figure 4). The control mice fed LFD had an average FBG level of 79 mg/dL. The consumption of HFD for 20 weeks resulted in a statistically significant increase in FBG in control mice: 165 mg/dL (*p* < 0.05) (Figure 4A). However, we detected lower FBG levels in HFD mice exposed to 25 and 50 ppm As: 128 and 117 mg/dL, respectively (Figure 4B,C). The differences in FBG between the HFD control and the HFD iAs-treated mice were statistically significant (*p* < 0.05). The exposure to iAs had no significant effects on FBG in the LFD group. For all mice, regardless of diet or iAs exposure, OGTT showed the characteristic rapid rise in blood glucose, peaking within 15–30 min of glucose challenge. This peak was followed by a gradual decrease, indicative of the uptake of glucose by liver and peripheral tissues. Blood glucose levels in LFD groups peaked at approximately 350 mg/dL and approached baseline levels by 120 min after glucose challenge, whereas HFD groups peaked at approximately 400 mg/dL and remained elevated compared with LFD groups (Figure 4A–C). To quantify glucose tolerance, we calculated the area under the OGTT curve (AUC) for all treatment groups. AUC values were significantly higher for mice fed HFD than for mice fed LFD regardless of the iAs exposure (Figure 4D). The average AUC values were higher for HFD mice exposed to 25 and 50 ppm As (35,315 and 38,625 units, respectively) compared with HFD controls (34,847 units). However, the differences did not reach statistical significance.

**Figure 5:**
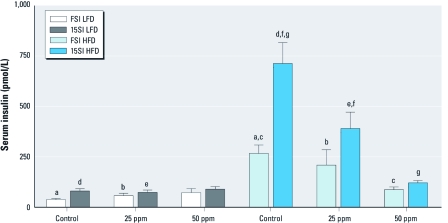
FSI and 15SI (mean ± SE) in control mice or mice exposed to 25 or 50 ppm As and fed LFD or HFD (*n* = 8–10). Values marked with the same letters are statistically significantly different: for b, c, and f, *p* < 0.05; for a, d, e, and g, *p* < 0.001.

**Figure 6:**
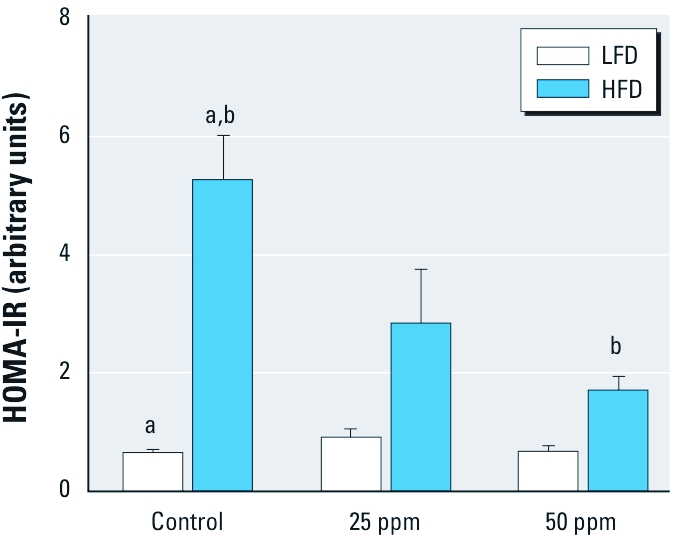
HOMA-IR values (mean ± SE) for control mice and mice exposed to 25 or 50 ppm As and fed LFD or HFD (*n* = 6–10). Values marked with the same letters are statistically significantly different: for b, *p* < 0.01; and for a, *p* < 0.001.

*Insulin resistance.* We measured FSI and serum insulin at 15 min into OGTT (15SI) to characterize insulin secretion in response to the glucose challenge and insulin resistance. Both FSI and 15SI levels were affected by HFD and exposure to iAs (Figure 5). In general, mice in the HFD groups had elevated FSI and 15SI levels compared with mice in the LFD groups. However, FSI and 15SI values in HFD mice decreased with iAs exposure in a concentration-dependent manner, resulting in near-normal values for HFD mice exposed to 50 ppm As. We found no statistically significant effects of iAs exposure on FSI or 15SI in the LFD group. We used FBG and FSI values to calculate HOMA-IR, an indicator of insulin resistance. The average HOMA-IR values were consistently higher in HFD than in LFD groups (Figure 6). iAs exposure did not change HOMA-IR in mice fed LFD, but it decreased HOMA-IR in the HFD group in a concentration-dependent manner, thus reducing the difference between the mice in HFD and LFD groups. The average HOMA-IR value was 5 times higher in control HFD than in control LFD mice, but only 3 times and 2.5 times higher in HFD versus LFD mice exposed to 25 and 50 ppm As, respectively.

**Figure 7:**
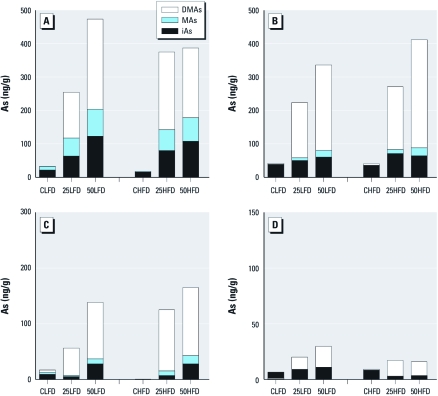
Speciation of As in tissues involved in regulation of glucose homeostasis. Mean concentrations of iAs, MAs, and DMAs are shown for liver (*A*), muscle (*B*), pancreas (*C*), and adipose tissue (*D*) from control mice (CLFD, CHFD) and mice exposed to 25 ppm As (25LFD, 25HFD) or 50 ppm As (50LFD, 50HFD) and fed LFD or HFD. For *A, B*, and *D*; *n* = 9–10; for *C*, *n* = 5–7.

*Tissue distribution of iAs metabolites.* We used HG-CT-AAS to determine concentrations of the metabolites of iAs in pancreas and in tissues involved in postprandial glucose metabolism, including liver, skeletal muscle, and fat (Figure 7). Exposure to iAs resulted in a concentration-dependent accumulation of iAs metabolites in the liver, skeletal muscle, pancreas, and fat of mice on LFD. We also found a similar concentration-dependent increase in iAs metabolite levels in the muscle and pancreas of HFD mice. In contrast, in HFD mice exposed to 25 ppm As, liver and fat were saturated with iAs metabolites, because the concentrations of these metabolites did not significantly increase after exposure to 50 ppm. For mice in the iAs-treated groups, we found the highest concentrations of iAs metabolites in the liver, followed by muscle, pancreas, and fat. DMAs was the major metabolite detected in the tissues of mice exposed to iAs. The tissues of control mice in both LFD and HFD groups contained mainly iAs.

## Discussion

Both male and female C57BL/6 mice have been frequently used in studies examining effects of obesogenic diets, including obesity-related type 2 diabetes. This mouse strain is characterized by a low baseline occurrence of diabetes and a high susceptibility to diet-induced diabetes (Petro et al. 2004; Surwit et al. 1988, 1995). Our previous work has shown that male C57BL/6 mice are also a good animal model for studying the diabetogenic effects of iAs exposure (Paul et al. 2007b). When exposed to iAs^III^ in drinking water and fed a regular, grain-based laboratory diet (LabDiet, Richmond, IN), these mice developed glucose intolerance characterized by abnormally high blood glucose levels after an intraperitoneal injection of glucose. However, the grain-based diet contained 19.5–28.6 ppb As (mainly iAs) and was a source of a relatively high background iAs exposure, possibly compromising the study design. To avoid this problem in the present study, we used only purified diets that did not contain grain components. The levels of As in these diets were below the detection limit of HG-CT-AAS for this type of matrix (~ 0.5 ppb). Additional information about the previously used grain-based diet and LFD and HFD used in this study is provided in Supplemental Material, Table 1 (doi:10.1289/ehp.1003324).

Our OGTT results showed that mice on the purified LFD, unlike mice on the grain-based diet (Paul et al. 2007b), did not develop glucose intolerance after exposure to iAs (Figure 4). When analyzing this unexpected result, we found that the mice fed the purified LFD or HFD drank less water than did mice in the previous study that were fed the grain-based diet [see Supplemental Material, Figure 2 (doi:10.1289/ehp.1003324)]. Consequently, the iAs intakes were significantly lower for the LFD and HFD mice in the present study, resulting in lower concentrations of As species in the target tissues. For example, the mice fed the grain-based diet that developed glucose intolerance after exposure to 50 ppm As (Paul et al. 2007b) drank, on average, 2.5 mL water per day (~ 125 μg/day As); liver and pancreas of these mice contained > 1,100 and 350 ng As/g, respectively. In comparison, the LFD mice exposed to 50 ppm As in the present study drank only 1.6 mL water per day, which represents a daily As intake of 81 μg; the average concentrations of As in the liver and pancreas were 474 and 138 ng/g, respectively. These data suggest that reaching critical threshold concentrations of As in tissues involved in the regulation of glucose homeostasis is essential for development of iAs-induced diabetes in mice on diets with low fat content. Thus, concentrations substantially > 50 ppm As would have to be used in the present study to reach the critical mass of As metabolites in the target tissues and to produce a diabetic phenotype in mice fed the purified LFD.

The main goal of our study was to characterize the diabetogenic effects of combined exposure to iAs and HFD. Specifically, we wanted to examine whether mice fed the obesogenic HFD are more susceptible to the diabetogenic effects of chronic exposure to iAs. Type 2 diabetes results from a progressive insulin secretory defect on the background of insulin resistance (American Diabetes Association 2006). Early stages of the disease are characterized by resistance of the liver and peripheral tissues to insulin signal, resulting in hyperglycemia, hyperinsulinemia, and glucose intolerance. Characteristics of advanced disease are failure of the pancreatic β cells to keep up with the increasing demand for insulin and a gradual decay of β-cell functions. Our findings of high FBG, FSI, 15SI, and HOMA-IR in the control obese mice fed HFD are consistent with the early stages of type 2 diabetes. Fat represented > 52% of body mass of the control HFD mice. In addition, TAG levels were significantly higher in the liver and plasma of these mice compared with the LFD controls. The concomitant exposure to iAs dramatically changed the phenotype associated with HFD consumption. The HFD mice exposed to 50 ppm As accumulated less fat (~ 30% of body mass) and had lower plasma TAG than the HFD controls. The FBG, FSI, 15SI, and HOMA-IR values were significantly lower in the HFD group exposed to 50 ppm As than in HFD controls. However, glucose intolerance measured by OGTT remained high and appeared even more pronounced than in HFD controls. The shape of the OGTT curve also changed, showing high blood glucose levels already at 15–30 min after the glucose dose. This time interval is consistent with postprandial uptake of glucose from portal circulation by the liver, indicating that hepatic metabolism of glucose may be affected by iAs exposure.

This is the first study to show that chronic exposure to iAs suppresses diet-induced obesity in laboratory mice. Data collected here do not provide information about mechanisms underlying the antiobesogenic effects of iAs. However, previous work has shown that iAs^III^ inhibits signal transduction mechanisms that are responsible for adipocyte differentiation (Trouba et al. 2000; Wauson et al. 2002). Differentiated adipocytes are responsible for fat (TAG) accumulation in adipose tissues. Thus, it is plausible that the limited fat accumulation in HFD mice exposed to iAs is due to the inhibition of adipocyte differentiation by iAs or by its trivalent methylated metabolites. An alternative mechanism could be associated with impaired TAG synthesis in the liver (e.g., as a result of hepatic insulin resistance) and decreased secretion of TAG for transport to the adipose tissues. The trend for lowering hepatic TAG accumulation and the decreased plasma TAG levels in the iAs-exposed mice on HFD are consistent with this mechanism.

Taken together, our data suggest that iAs exposure may act synergistically with HFD-induced obesity, producing glucose intolerance in mice with a relatively low adiposity. Notably, the diabetic phenotype associated with the exposure of HFD mice to iAs differs from that of typical type 2 diabetes. It combines pronounced glucose intolerance and a moderately elevated FBG with almost normal insulin sensitivity when measured by FSI, 15SI, or HOMA-IR. Low FSI, 15SI, and HOMA-IR values are generally interpreted as indicators of adequate insulin sensitivity. However, low FSI and 15SI values can also indicate impaired insulin production by β cells. With low FSI, the calculated HOMA-IR value would also be low, assuming that FBG does not increase by a significant margin. At the same time, impaired insulin production would result in a pronounced glucose intolerance in a manner that is consistent with the phenotype described for the HFD mice exposed to 50 ppm As. Recent laboratory studies have shown that low, subtoxic concentrations of iAs^III^ can inhibit glucose-stimulated insulin expression or secretion by cultured β cells (Díaz-Villaseñor et al. 2006) and a rat insulinoma (INS-1) cell line (Fu et al. 2010). Thus, it is possible that both insulin resistance in the liver and peripheral tissues and impaired β-cell function determine the diabetic phenotype produced by the combined exposure to iAs and HFD. Alternatively, iAs exposure could interfere with the production or signaling of the incretin hormones glucose-dependent insulinotropic polypeptide (GIP) and glucagon-like peptide-1 (GLP-1) that, respectively, induce insulin secretion from the pancreatic β cells and suppress glucagon release from the α cells during meals.

Using HOMA-IR instead of a direct measurement of β-cell function and signals regulating this function represents a significant limitation for this study. Additional and more comprehensive methods (e.g., insulin tolerance test, frequently sampled intravenous glucose tolerance test, or the hyperinsulinemic euglycemic clamp) will be needed in future studies to pinpoint the exact mechanisms of the diabetogenic effects of iAs exposure alone and combined with diet-induced obesity.

## Supplemental Material

(108 KB) PDF

## References

[r1] American Diabetes Association (2006). Standards of medical care in diabetes—2006.. Diabetes Care.

[r2] Díaz-Villaseñor A, Sánchez-Soto MC, Cebrián ME, Ostrosky-Wegman P, Hiriart M (2006). Sodium arsenite impairs insulin secretion and transcription in pancreatic β-cells.. Toxicol Appl Pharmacol.

[r3] Fu J, Woods CG, Yehuda-Shnaidman E, Zhang Q, Wong V, Collins S (2010). Low-level arsenic impairs glucose-stimulated insulin secretion in pancreatic beta cells: involvement of cellular adaptive response to oxidative stress.. Environ Health Perspect.

[r4] Hernández-Zavala A, Matoušek T, Drobná Z, Adair BM, De˘dina J, Thomas DJ (2008). Speciation analysis of arsenic in biological matrices by automated hydride generation-cryotrapping-atomic absorption spectrometry with multiple microflame quartz tube atomizer (multiatomizer).. J Anal At Spectrom.

[r5] IARC (International Agency for Research on Cancer) (1987). Overall Evaluations of Carcinogenicity: An Updating of IARC Monographs Volumes 1 to 42.. IARC Monogr Eval Carcinog Risks Hum Suppl.

[r6] Mazumder DN (2005). Effect of chronic intake of arsenic-contaminated water on liver.. Toxicol Appl Pharmacol.

[r7] Navas-Acien A, Silbergeld EK, Streeter RA, Clark JM, Burke TA, Guallar E (2006). Arsenic exposure and type 2 diabetes: a systematic review of the experimental and epidemiological evidence.. Environ Health Perspect.

[r8] Paul DS, Harmon AW, Devesa V, Thomas DJ, Styblo M (2007a). Molecular mechanisms of diabetogenic effects of arsenic: inhibition of insulin signaling by arsenite and methylarsonous acid.. Environ Health Perspect.

[r9] Paul DS, Hernández-Zavala A, Walton FS, Adair BM, De˘dina J, Matoušek T (2007b). Examination of the effects of arsenic on glucose homeostasis in cell culture and animal studies: development of a mouse model for arsenic-induced diabetes.. Toxicol Appl Pharmacol.

[r10] Petro AE, Cotter J, Cooper DA, Peters JC, Surwit SJ, Surwit RS (2004). Fat, carbohydrate, and calories in the development of diabetes and obesity in the C57BL/6J mouse.. Metabolism.

[r11] Surwit RS, Feinglos MN, Rodin J, Sutherland A, Petro AE, Opara EC (1995). Differential effects of fat and sucrose on the development of obesity and diabetes in C57BL/6J and A/J mice.. Metabolism.

[r12] Surwit RS, Kuhn CM, Cochrane C, McCubbin JA, Feinglos MN (1988). Diet-induced type II diabetes in C57BL/6J mice.. Diabetes.

[r13] Trouba KJ, Wauson EM, Vorce RL (2000). Sodium arsenite inhibits terminal differentiation of murine C3H 10T1/2 preadipocytes.. Toxicol Appl Pharmacol.

[r14] Walton FS, Harmon AW, Paul DS, Drobna Z, Patel YM, Styblo M (2004). Inhibition of insulin-dependent glucose uptake by trivalent arsenicals: possible mechanism of arsenic-induced diabetes.. Toxicol Appl Pharmacol.

[r15] Wauson EM, Langan AS, Vorce RL (2002). Sodium arsenite inhibits and reverses expression of adipogenic and fat cell-specific genes during in vitro adipogenesis.. Toxicol Sci.

