# Supplementary material for: Characterization of the Impaired Glucose Homeostasis Produced in C57BL/6 Mice by Chronic Exposure to Arsenic and High-Fat Diet
Source: Environ Health Perspect. 2011 May 18;119(8):1104–9. doi: 10.1289/ehp.1003324 (PMC3237360; doi:10.1289/ehp.1003324)
Supplement: (108 KB) PDF [file ehp.1003324.s001.pdf]

SUPPLEMENTAL MATERIAL

Characteristics of the Impaired Glucose Homeostasis Produced in C57BL/6 Mice by Chronic Exposure to Arsenic and High-Fat Diet

David S. Paul, Felecia S. Walton, R. Jesse Saunders, Miroslav Stýblo

Department of Nutrition, University of North Carolina at Chapel Hill

Chapel Hill, North Carolina, USA

Table of Content

Supplemental Material, Table 1: Basic characteristics of the mouse diets ………………. 2
used in this and previous studies

Supplemental Material, Figure 1: Hematocrits of mice exposed to 25 ppm or
50 ppm As and control mice fed LFD or HFD ……………………………………..…. 3

Supplemental Material, Figure 2: Effects of diet composition on water consumption
and arsenic intake by control mice and mice exposed
to 25 or 50 ppm As in drinking water …………………………………………………………. 4

Supplemental Material, Table 1: Basic characteristics of the purified diets used in this study and the grain-based diet used in previous studies[1]

Diet	Purified Low-Fat Diet (LFD)	Purified High-Fat Diet (HFD)	Grain-based diet
Manufacturer	Research Diets, Inc.	Research Diets, Inc.	LabDiet PMI Nutrition International
Description	11 kcal% fat w/sucrose Surwit Diet (D12329)	58 kcal% fat w/sucrose Surwit Diet (D12331)	PicoLab Mouse Diet 20 (No. 5058)
Energy density (kcal/g)	4.07	5.56	3.75
Fat (% kcal/g)	10.5	58.0	21.6
Protein (% kcal/g)	16.4	16.4	23.2
Carbohydrate (% kcal/g)	73.1	25.5	55.1

[1] Paul DS, Hernández-Zavala, A, Walton, FS, Adair, BM, Dědina, J, Matoušek, T, et al. 2007. Examination of the effects of arsenic on glucose homeostasis in cell culture and animal studies: Development of a mouse model for arsenic-induced diabetes. Toxicol Appl Pharmacol 222:305-314.

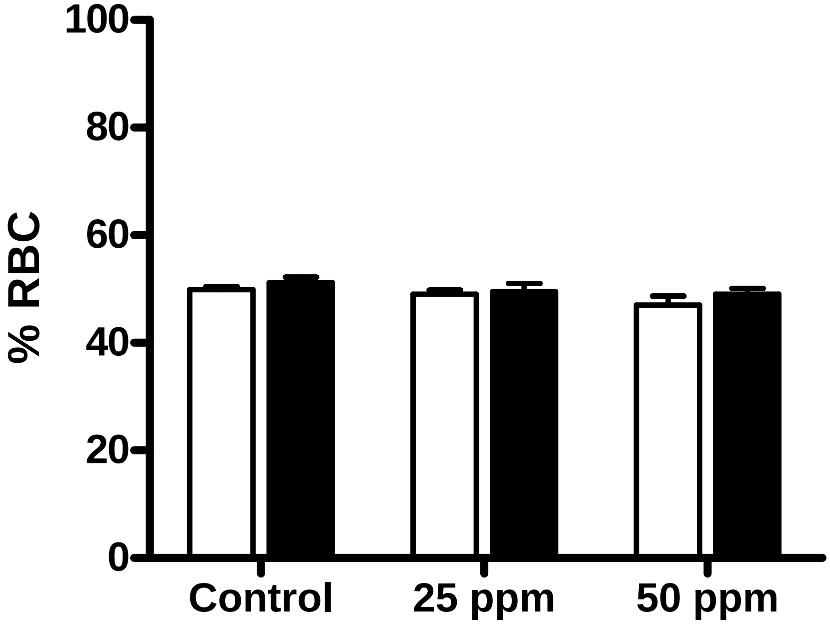

Supplemental Material, Figure 1: Hematocrits of mice exposed to 25 ppm or 50 ppm As or control mice fed LFD (□) or HFD (■). (Mean ± SEM, n = 5-10)

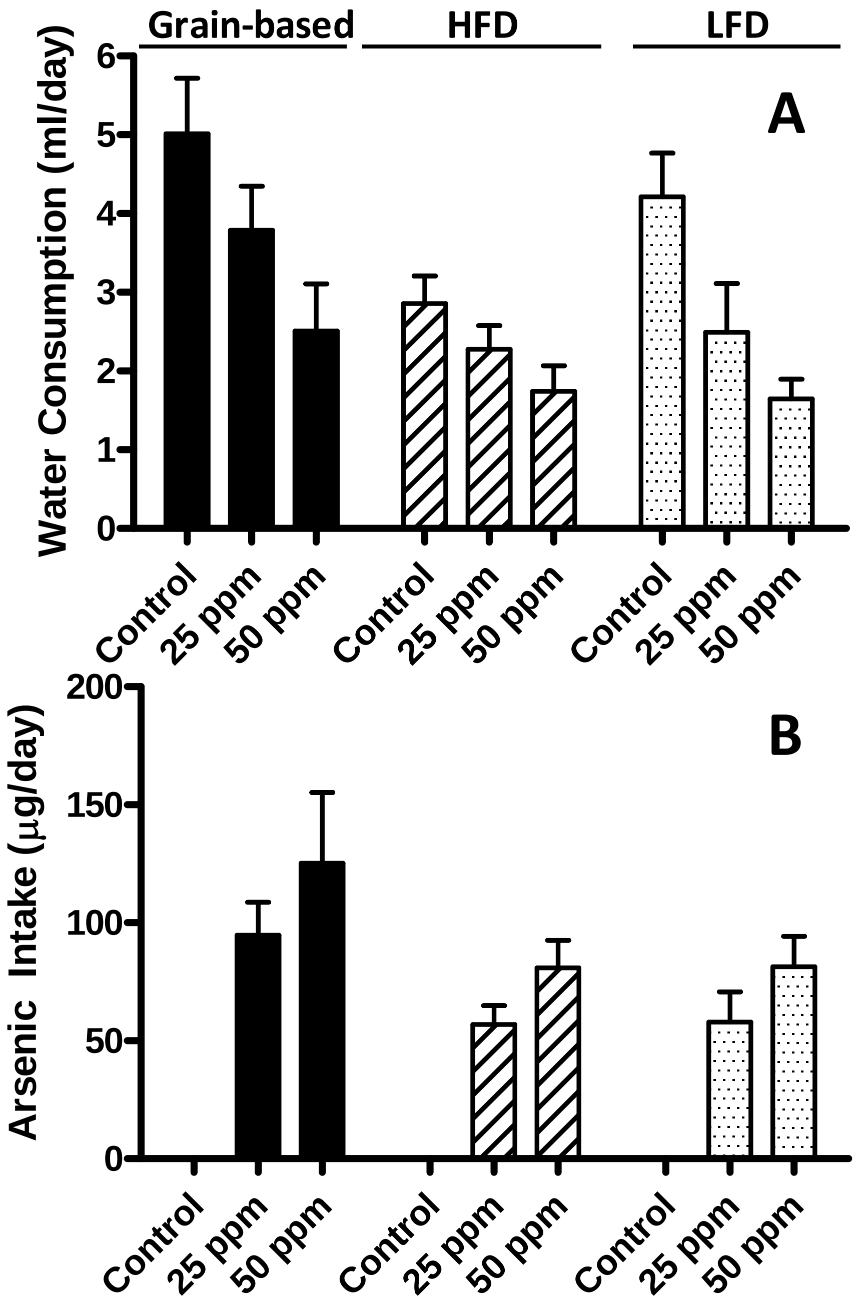

Supplemental Material, Figure 2: Effects of diet composition on water consumption (A) and arsenic intake (B) by control mice and mice exposed to 25 or 50 ppm As in drinking water. Results for mice fed a low fat grain-based diet[1] (Lab Diet 5058, Nutrition International, Brentwood, MO) and purified LFD and HFD are shown (Mean ± SEM, n = 15 for grain-based diet, n = 38 for HFD and LFD)

[1] Paul DS, Hernández-Zavala, A, Walton, FS, Adair, BM, Dědina, J, Matoušek, T, et al. 2007. Examination of the effects of arsenic on glucose homeostasis in cell culture and animal studies: Development of a mouse model for arsenic-induced diabetes. Toxicol Appl Pharmacol 222:305-314.
